# A Prompt to the Web: The Media and Health Information Seeking Behaviour

**DOI:** 10.1371/journal.pone.0034314

**Published:** 2012-04-03

**Authors:** Marie-Clare B. Hogue, Evan Doran, David A. Henry

**Affiliations:** 1 School of Medicine and Public Health, University of Newcastle, Callaghan, New South Wales, Australia; 2 The Hunter Valley Research Foundation, Maryville, New South Wales, Australia; 3 Institute for Clinical Evaluative Sciences, Toronto, Ontario, Canada; University of British Columbia, Canada

## Abstract

**Objective, Design, Setting and Participants:**

The objective was to investigate media influence on consumers' health related behaviours. A cross-sectional survey of randomly selected adults (18+ years) residing in the Hunter Region of New South Wales Australia was conducted. The sample was selected using a combination of the white pages and random digit dialling.

**Main Outcome Measures:**

The proportions of respondents who recalled seeing or hearing about conditions or treatments in the media over the 12 months prior to interview (August 2009–August 2010) and their subsequent health related behaviour.

**Results:**

Although most survey participants reported seeking health information from their doctors, around two-thirds of survey participants (551, 68.8%) recalled hearing, seeing or reading about one or more medical conditions (total = 1097 instances) in the mainstream media over the past 12 months. Almost 40% of respondents (307, 38.4%) stated that they had looked for more information about a condition as a result of hearing about it in the media, and most used the internet (269, 87.4%). More than a quarter of respondents (215, 26.9%) indicated that they had asked their doctor about a condition they had heard about in the media. Around half of those who asked their doctor (109, 50.6%) reported that their inquiry resulted in them receiving treatment, of whom almost half (53, 48.3%) reported being prescribed a medicine.

**Conclusion:**

The survey results show that consumers become aware of medicines through traditional media and then to learn more often turn to the internet where quality of information may be poor. (252 words)

## Introduction

The use of medicines in Australia is guided by the National Medicines Policy (NMP) a key priority of which is to promote “informed and active consumers” [1]. It is hoped that improved ‘medicines health literacy’ will help with consumer compliance, reduce adverse events and result in better health outcomes from medicine use overall [2]. Ensuring consumers are well-informed requires that people have access to and use good quality information about medicines and the conditions they address.

The public receives medicines information from a wide array of sources, including health professionals, lay ‘experts’, governments, patient organisations, and drug manufacturers. These provide information, advice and promotion, through various mediums – inter-personal communication, consumer medicines information, media reports, and internet web-sites [3]. Information quality from any source can be an issue but there is considerable ambivalence about the role of the media (e.g. television news and magazines) in informing people about medicines. A reliance on the media for medicine related information is considered problematic because of doubts about accuracy, balance and the influence of undeclared conflicts of interest [4].

Although it is generally acknowledged that the media can usefully raise awareness about a medicine or its associated condition, there is long-standing concern over inaccurate and sensational reporting and the presence of biased drug promotion in the media [5]–[7]. Under current policy direct-to-consumer advertising of prescription medicines is banned in Australia, however manufacturers are able to indirectly promote their products to the public via disease awareness advertising e.g. ‘ask your doctor’ advertisements. Assuming people vary in their capacity to critically appraise medicines information, poor reporting and promotional spin may misinform consumers about the risks of a condition and the benefits of treatment [8], [9].

Misinformed consumers may have heightened concern and/or expectations, which may in turn lengthen and complicate medical consultations, generate inappropriate requests for treatment and possibly result in ‘sub-optimal’ medicines use with unnecessary costs and avoidable adverse effects [9], [10]. These concerns have been compounded by the rising popularity of the web and more recently the rise of social media such as Facebook, YouTube and Twitter [11]–[13].

Although concern about the role of the media in informing people about medicines and conditions is widespread [13] there has been little study of how consumers interact with the media in learning about medicines. There is a little doubt that the media often raises interest in, and action regarding, a condition or treatment. But how common is it for people to respond to news of a condition or treatment by seeking further information? Where do they go? How often do they talk to their doctor? How often do they turn to the internet? Answering such questions is important for policies designed to achieve well informed consumers [6].

As part of a larger study looking at the medicines information environment in Australia [14], we surveyed a sample of residents and asked them about what happens when they hear or read news about a condition or treatment in the media.

## Methods

### The survey

We conducted a cross sectional survey of randomly selected adults (18+ years) residing in the Hunter Region of New South Wales Australia. We conducted a cross sectional survey of randomly selected adults (18+ years) residing in the Hunter Region of New South Wales Australia. Australian census data shows the population of the Hunter to be broadly similar to the Australian population [15]. The survey was conducted over 4 weeks between 17 August and 17 September 2010. Eight hundred interviews were sought with equal numbers males and females. All data were collected using a Computer-Assisted Telephone Interview (CATI). Sampling involved a two stage randomisation selection process. In the first stage the CATI program, randomly selected a household using a combination of electronic white pages and random digit dialling. In the second stage, the number of eligible persons in the household was identified, each person assigned a number with CATI program then randomly selecting one person as the respondent. Once identified, the selected respondent was not substituted with other household members. Up to 11 call attempts were made to each household to complete an interview.

### Questionnaire

The questionnaire contained several distinct exercises related to different aspects of the larger study. The results reported here relate to open-ended questions exploring the individual's response to hearing about a medical condition in the media. The questions used general vernacular phrases such as ‘the media’ to encompass all particular media forms (e.g. television news, print news) and likewise ‘heard about’ to cover seen or read. We sought data on the range of media sources people identify as bringing their attention to a condition or treatment; whether people seek further information about a condition or treatment they have become aware of; what sources of information do they use; and whether their inquiry results in them receiving treatment. We also asked respondents whether hearing about conditions in the media caused them to worry about their health. To avoid people having to reveal intimate personal information we did not ask people to identify the condition or treatment they inquired further about. In addition, we collected demographic information such as gender, age, educational achievement, employment status, self-reported health (poor to excellent), whether or not the participant had private health insurance.

### Data analysis

The open-ended questions sought descriptive data requiring minimal interpretation; responses were categorised by a single analyst (MCH). Categorised responses are presented as the percentage of respondents nominating each category (respondents could provide more than one answer to a question therefore the number of responses may exceed 100%).

Descriptive statistics were used to summarise the data on reported behaviours. Further analysis centred on the differences in reported behaviours by gender, age and education with associations explored using Pearson chi square analysis and odds ratios (OR) with their 95% confidence intervals. Only statistically significant associations are reported. All (demographic) data were de-identified and weighted to reflect the household size, age and gender distribution of the Hunter Region population based on the 2006 Census of Population and Housing [12].

The study was approved by the Human Research Ethics Committee of the University of Newcastle, Australia.

## Results

We surveyed a random sample of 800 residents of the Hunter region (412 female and 388 male), at a response rate of 69.7% (the proportion of people successfully contacted who agreed to participate). Table 1 summarises the characteristics of respondents.

**Table 1:** Respondent Characteristics.

Characteristic	HunterSample N = 800
**Gender**	
Male	388 (48.5%)
Female	412 (51.5%)
**Age**	
Younger 18–49 years	437 (54.6%)
Older 50+ years	373 (45.4%)
**Educational achievement**	
Secondary only	549 (68.6%)
Tertiary	251 (31.4%)
**Employment**	
Paid full-time/part time employment	456 (57%)
Not in labour force	344 (43%)
**Self-Rated Health**	
Poor/Fair	129 (16.1%)
Good, Very Good, Excellent	671 (83.9%)

Notes: The Hunter proportions are weighted to the population of the Hunter according to the 2006 Australian Bureau of Statistics Census. Self- assessed health status is weighted according to the Australian Bureau of Statistics National Health Survey 2007–2008.

### Usual sources for information

Respondents were asked the open-ended question – “where would you usually gain information about a medical condition?” Table 2 lists the nominated sources. The four most frequently cited were doctors (650, 81.3%) the internet (338, 42.2%) the media (112, 14%) and family or friends (94, 11.8%). Smaller proportions of respondents reported a range of other sources of information. Also using an open-ended question, respondents were asked – “where would you usually gain information about a treatment?” The majority reported their doctor (678, 84.8%) followed by the internet (260, 32.6%) and pharmacist (98, 12.2%) with smaller proportions reporting the use of other sources of information.

**Table 2:** Usual sources of information.

Source	Usually seek information about a condition N = 800	Usually seek information about a treatment N = 800
Doctor	650 (81.3%)	678 (84.8%)
Internet	338 (42.2%)	260 (32.6%)
Media (TV, Radio, Print)	112 (14%)	79 (9.9%)
Family members or Friends	94 (11.8%)	51 (6.4%)
Chemist/pharmacist	51 (6.4%)	98 (12.2%)
Books/Journal articles	46 (5.7%)	37 (4.6%)
Allied or other health professional	31 (3.9%)	19 (2.4%)
Advertisements	25 (3.1%)	13 (1.6%)
Other	7 (0.9%)	7 (0.9%)

### Hearing about conditions and treatment in the media

Respondents were asked “Can you recall hearing or reading about any conditions in the media over the past 12 months.” Around two-thirds of survey participants (551, 68.8%) recalled hearing, seeing or reading about one or more medical conditions (total = 1097 instances) in the media over the past 12 months. Men were less likely than women to recall hearing about a medical condition (OR 0.61, 95% CI 0.45, 0.82). The five most commonly recalled sources (listed in Table 3) were television news (414, 75.1%), followed by print media (223, 40.1%), various forms of advertising (134, 24.3%), radio news (83, 15%) and the internet (45, 8.2%). The ten most commonly recalled conditions were cancer (354, 64.3%) diabetes (155, 28.1%), heart disease/stroke (147, 26.7%), obesity (88, 16.0%), Swine Flu (60, 10.9%), mental illness (including depression, bipolar disorder, anxiety) (50, 9.0%), common cold and flu (24, 4.3%), asthma (22, 4.0%) and osteoporosis/fracture (19, 3.5%). In the majority of instances (968, 88.2%) the conditions had been heard about before.

**Table 3:** Sources for hearing about a condition.

Source	Heard of a condition(N = 551)
Television news	414 (75.1%)
Print news (newspaper, magazine)	223 (40.4%)
Any advertising (TV/Radio/Print/Outdoor)	134 (24.3%)
Radio news	83 (15.0%)
Internet	45 (8.2%)
Family members or Friends	28 (5.1%)
Doctor, Pharmacist other Health Professional	21 (3.8%)
Other (e.g. Books, Journals)	16 (2.9%)

When asked “do you recall if any treatments were mentioned when you heard about the condition” in around two-thirds of instances (666, 60.7%) a treatment was also recalled as being mentioned in association with the condition. Respondents were asked the open-ended question – what treatment was mentioned?’ Responses were categorised (listed in Table 4) with the five most commonly recalled ‘treatments’ being lifestyle change i.e. diet, exercise and smoking cessation (192, 28.8%), prescription medication (128, 19.2%), vaccines (81, 12.2), chemo/radiotherapy (62, 9.3%) and screening/testing (56, 8.45).

**Table 4:** Recall a treatment being mentioned.

Treatments	YesN = 666
Lifestyle change	192 (28.8%)
Prescription medication	128 (19.2%)
Vaccine	81 (12.2%)
Chemo/radio therapy	62 (9.3%)
Monitor condition/screening/testing	56 (8.4%)
Surgery	45 (6.7%)
OTC medication/natural remedy	25 (3.7%)
Medical device	23 (3.4%)
See your doctor	18 (2.7%)
Unsure	14 (2.1%)
More research needed	8 (1.2%)
Gene therapy	7 (1.05%)
Psychological therapy/counselling	6 (0.9%)

In a separate question, respondents were asked if they recalled hearing about a treatment in the media in previous 12 months. Those that did recall hearing of a treatment were open-endedly asked to name the treatment. A total of 236 (29.5%) respondents recalled hearing of a treatment with most frequently recalled ‘treatment's being prescription medications (46, 19.5%), chemo/radio therapy (36, 15.3%), lifestyle change (38, 13.3%) and surgery (28, 12.1%). Just over three in ten respondents could not recall the type of treatment they had heard about.

### Seeking further information about conditions and treatments as a result of media attention

Respondents were asked “have you ever looked for more information about a medical condition that you had heard about in the media”, and if so, “where did you look.” Almost 40% of respondents (307, 38.4%) stated that they had looked. As shown in Table 5, by far the most commonly reported source was the internet (269, 87.4%), followed by books (23, 11.2%) and then the doctor (17, 8.3%). There was a statistically significant difference on only one demographic variable; those with post-secondary school education were more likely to report seeking further information (OR 1.66, 95%CI 1.21–2.28). The majority (280 91.1%) of those who looked for information about a condition felt that the information they found was helpful.

**Table 5:** Sources of further information.

Source	Sought more information about a treatment(N = 205)	Sought more information about a condition(N = 307)
Internet	168 (81.9%)	269 (87.4%)
Other (e.g. Books, Journals)	23 (11.6%)	37 (11.9%)
Doctor, Pharmacist or health professional	17 (8.1%)	14 (4.6%)
Print news (newspaper, magazine)	2 (1.4%)	5 (1.6%)
Family members or Friends	2 (1.2%)	3 (1.0%)
Any advertising TV/Radio/Print/Outdoor	2 (1.0%)	0

Approximately one-quarter (205, 25.6%) of respondents stated that they had looked for further information about a treatment they heard about in the media of whom (168, 81.9%) report using the internet (see Table 5). Two demographic characteristics showed a statistically significant difference: men were less likely than women to report having looked (21.4% versus 25.6%; OR 0.64, 95% CI 0.46, 0.89); and those with post-secondary education were more likely to report look for more information (27.5% versus 20.5% OR 1.6, 95%CI 1.1–2.3) Almost half (98, 47.7%) of those who looked for information about a treatment indicated that the treatment was a prescription drug. The majority (183, 89.2 .1%) of those who looked for information about a treatment felt that the information they found was helpful.

### Consequences of exposure to information in the media regarding conditions and treatments

Respondents were asked specifically “have you ever asked your doctor about a medical condition that you have heard about in the media?” More than a quarter of respondents (215, 26.9%) indicated that they had. Those with post-secondary education were more likely to report asking their doctor about a medical condition (29.6% versus 22.2%; OR 1.76, 95% CI 1.23–2.52). Around half of the 215 who asked their doctor about a condition (109, 50.6%) reported that their inquiry resulted in them receiving treatment, of whom almost half (53, 48.3%) reported being prescribed a medicine.

Respondents were asked specifically whether they had asked their doctor about a treatment they had heard about in the media with almost a quarter of respondents (189, 23.6%) indicated that they had. Men were less likely than women to report asking their doctor (18.5 versus 28.4; OR 0.58, 95% CI 0.41, 0.80). Of the 189, who asked their doctor about a treatment, 85 (44.9%) reported that this had resulted in them receiving treatment. Of those reporting receiving a treatment, over half (49, 58.1%) reported receiving a prescription medicine. Respondents were also asked whether they had asked a doctor about a ‘brand name’ drug they heard about in the media. One-sixth of respondents (120, 15.0%) indicated that they had and of these respondents over a third (45, 37.9%) reported being prescribed the drug.

In a separate question respondents were asked whether they had ever heard about a condition in media that had caused them to worry about it. One-hundred and fifty (18.7%) respondents indicated that they had, with 87 of these (58%) reporting going on to seek advice from their doctor about the condition and of these 35 (40.5%) indicated that this had resulted in treatment.

## Discussion

The survey results show exposure to information about medical conditions and associated treatments in the broadcast and print media often prompts consumers to seek more information, predominantly on the internet. The results also show that seeking further information often results in consumers requesting or receiving a medicine or other forms of ‘treatment’ – a category that for our respondents included ‘life-style changes’ such as diet and exercise as well as what would be more conventionally regarded as treatments such as prescription medicines.

The majority of respondents recalled hearing about a condition or treatment in the media. Numerous illnesses were recalled but the prominence of cancer is in line with findings of other studies that suggest both its salience in people's thinking about illness and also that cancer is a mainstay of news and commentary on health and illness [16]. Many respondents also recalled hearing of numerous types of treatments in the media. Interestingly most respondents identified lifestyle changes such as diet, exercise and stopping smoking as the treatments the ‘treatment’ they recalled hearing about. This probably reflects the prominence of public health promotion campaigns at the time of survey. For example, in addition to the long-running anti-smoking media campaign, a high profile anti-obesity media campaign was being run in the Hunter (as elsewhere in NSW). Health promotion advocates might take some satisfaction in the high frequency of reported recall of such ‘treatments’ in our survey.

As in other consumer surveys [17], [18] most respondents nominated their doctor as their usual source of information about a condition or treatment. News of a condition or treatment prompted many respondents to talk to their doctor with around half of these reporting this resulting in them receiving a treatment and for half of these the treatments were prescription medicines. Hearing about a specific brand name drug in the media also prompted discussion with a doctor and this frequently resulted in the medicine being prescribed. Our data cannot show whether the media prompted a helpful discussion between doctor and patient with appropriate treatment (prescription medicine or otherwise) being prescribed. In the absence of data to the contrary, we might assume that in each case the media prompted a helpful discussion between doctor and patient and appropriate treatment being prescribed. However, that media reports are cited as prompting consumers to speak to their doctor underscores the importance for balance and accuracy in media reports of conditions and treatments to avoid doctors having to correcting misapprehensions and heightened expectations [9].

While doctors featured prominently, for many respondents the doctor is not the first or only port of call for further information of a condition or treatment heard about in the media. Among those who looked for more information, the internet was overwhelmingly the most frequently reported source. These results support the findings of other studies that suggest that the internet is becoming a key source of health related information in the general population [19]–[23]. Our results cannot show how respondents searched for information, or what web sites they visited but our data does show most were satisfied with the information they found on the internet with most indicating that they found it helpful. Again, in the absence of data to the contrary we might assume that satisfaction indicates helpful exposure to quality information. Satisfaction notwithstanding, given the uneven quality of information health information on the internet it could equally be possible that respondents have been exposed to poor quality information [24].

The results show an interesting difference between where respondents reportedly usually seek information - their doctor; and where they will turn on hearing about a condition or treatment in the media – the internet. The difference possibly reflects a degree of social desirability bias, where respondents anticipate that reporting the doctor as their usual source is the most appropriate response. Equally, the difference might reflect the increasing ease and immediacy of Australians being able to use the internet as a source of health and medicines information.

The internet has become a vast repository of both technical information and non-technical health information [22] that may empower people to maintain and improve their own health and the health of those around them [20]. The potential for the internet to provide sound medicines information was demonstrated in a recent Australian government campaign aimed at consumers (‘Use Medicines Wisely’) which used the internet, Twitter and Facebook [25]. Benefits aside, concern about the quality of medicines information available via the internet is growing [26]. The quality of the medicines information available to Australian consumers via web pages and blogs has long been questioned [27]. and questions have multiplied with the rise of social media, not the least because of their potential as platforms for drug marketing or ‘e-detailing’ [10], [11].

Our survey results confirm the role of television and print media as important influences on health information seeking behaviour. The data indicate that consumers respond to news of a condition or a treatment by talking to trustworthy sources such as their doctor or other health professional. Most significantly, the data also indicate that media reports act as a launch pad to the increasingly reachable internet where the quality of information is variable and drug promotion increasingly prevalent. An implication for current Australian medicines policy is the need to recognise that the traditional media, the internet and social media are increasingly integrated; media reports need to comply with current regulations and restrictions to minimise the potential for inappropriately prompting consumers to the less regulated web.

Our study has a number of limitations. The open-ended questions required people to recall past events and behaviours. The results are based on self-report, not observed behaviour. The survey was conducted in a single region – the Hunter region of New South Wales. However, while there are some demographic differences between the Hunter and other Australian regions, there is no reason to expect responses to be substantially different elsewhere in Australia.

### Conclusion

Keeping consumers well-informed about medicines has become more challenging as the sources of information have proliferated. Our survey shows that consumers become aware of medicines through traditional media and then often turn to the internet for further information. There are long-standing concerns about the quality of information in the traditional media and growing concern about the quality of information available on the internet and the newer social media. Inaccurate or sensational reporting in the traditional media may be compounded by people seeking further information on the internet and being exposed to even more inaccurate or biased information. Increased complexity and integration of the medicines information environment calls for a policy that can provide a framework for the coordination of medicines information through all media.
